# Supplementary material for: A comparison of comorbidity measures for predicting mortality after elective hip and knee replacement: A cohort study of data from the National Joint Registry in England and Wales
Source: PLoS One. 2021 Aug 12;16(8):e0255602. doi: 10.1371/journal.pone.0255602 (PMC8360555; doi:10.1371/journal.pone.0255602)
Supplement: S1 Table — (DOCX) [file pone.0255602.s001.docx]

S1 Table: ASA Grade and comorbidity scores for the study sample of people having a primary THR and KR

| Characteristic | THR | KR |
| --- | --- | --- |
|  | N = 276,594^1^ | N = 338,287^1^ |
| **ASA Grade** |  |  |
| I | 36,119 (13%) | 31,947 (9.4%) |
| II | 194,358 (70%) | 249,759 (74%) |
| III | 44,821 (16%) | 55,562 (16%) |
| IV +V | 1,296 (0.5%) | 1,019 (0.3%) |
| **CCI (original)** |  |  |
| Primary episode |  |  |
| 0 | 189,453 (68%) | 219,156 (65%) |
| 1 | 59,849 (22%) | 84,191 (25%) |
| 2 | 17,896 (6.5%) | 23,841 (7.0%) |
| 3+ | 9,396 (3.4%) | 11,099 (3.3%) |
| 1-year lead-up |  |  |
| 0 | 182,638 (66%) | 210,718 (62%) |
| 1 | 60,985 (22%) | 85,772 (25%) |
| 2 | 20,451 (7.4%) | 27,110 (8.0%) |
| 3+ | 12,520 (4.5%) | 14,687 (4.3%) |
| 2-year lead-up |  |  |
| 0 | 177,562 (64%) | 203,726 (60%) |
| 1 | 61,364 (22%) | 86,534 (26%) |
| 2 | 22,537 (8.1%) | 29,997 (8.9%) |
| 3+ | 15,131 (5.5%) | 18,030 (5.3%) |
| 5-year lead-up |  |  |
| 0 | 168,853 (61%) | 191,873 (57%) |
| 1 | 61,666 (22%) | 86,846 (26%) |
| 2 | 26,109 (9.4%) | 35,269 (10%) |
| 3+ | 19,966 (7.2%) | 24,299 (7.2%) |
| All episodes |  |  |
| 0 | 157,817 (57%) | 178,179 (53%) |
| 1 | 61,079 (22%) | 86,097 (25%) |
| 2 | 31,367 (11%) | 41,565 (12%) |
| 3+ | 26,331 (9.5%) | 32,446 (9.6%) |
| **CCI (SHMI)** |  |  |
| Primary episode | 0.0 (0.0, 4.0), max=52.0 | 0.0 (0.0, 4.0), max=60.0 |
| 1-year lead-up | 0.0 (0.0, 4.0), max=58.0 | 0.0 (0.0, 4.0), max=61.0 |
| 2-year lead-up | 0.0 (0.0, 4.0), max=65.0 | 0.0 (0.0, 4.0), max=77.0 |
| 5-year lead-up | 0.0 (0.0, 4.0), max=70.0 | 0.0 (0.0, 4.0), max=83.0 |
| All episodes | 0.0 (0.0, 7.0), max=75.0 | 0.0 (0.0, 7.0), max=83.0 |
| **Elixhauser** |  |  |
| Primary episode | 0.0 (0.0, 1.0), max=32.0 | 0.0 (0.0, 1.0), max=36.0 |
| 1-year lead-up | 0.0 (0.0, 3.0), max=42.0 | 0.0 (0.0, 2.0), max=43.0 |
| 2-year lead-up | 0.0 (0.0, 3.0), max=46.0 | 0.0 (0.0, 3.0), max=43.0 |
| 5-year lead-up | 0.0 (0.0, 3.0), max=46.0 | 0.0 (0.0, 3.0), max=54.0 |
| All episodes | 0.0 (0.0, 3.0), max=53.0 | 0.0 (0.0, 3.0), max=54.0 |
| **Frailty** |  |  |
| Primary episode | 0.0 (0.0, 1.3), max=28.6 | 0.0 (0.0, 1.1), max=27.4 |
| 1-year lead-up | 0.0 (0.0, 1.5), max=46.1 | 0.0 (0.0, 1.5), max=46.0 |
| 2-year lead-up | 0.0 (0.0, 1.8), max=61.3 | 0.0 (0.0, 1.8), max=71.3 |
| 5-year lead-up | 0.5 (0.0, 2.3), max=79.4 | 0.7 (0.0, 2.3), max=93.3 |
| All episodes | 1.3 (0.0, 3.2), max=79.4 | 1.5 (0.0, 3.4), max=93.3 |
| ^1^Statistics presented: n (%); median (IQR), max | | |
