# Supplementary material for: A comparison of comorbidity measures for predicting mortality after elective hip and knee replacement: A cohort study of data from the National Joint Registry in England and Wales
Source: PLoS One. 2021 Aug 12;16(8):e0255602. doi: 10.1371/journal.pone.0255602 (PMC8360555; doi:10.1371/journal.pone.0255602)
Supplement: S2 Table — (DOCX) [file pone.0255602.s002.docx]

S2 Table: A comparison of the comorbidity scores of people having a primary THR who died within 90-days of their operation and those who were alive at 90-days

| Characteristic | **Alive at 90 days** | **Died by 90 days** |
| --- | --- | --- |
|  | N = 275,640^1^ | N = 954^1^ |
| **ASA Grade** |  |  |
| I | 36,089 (13%) | 30 (3.1%) |
| II | 193,934 (70%) | 424 (44%) |
| III | 44,379 (16%) | 442 (46%) |
| IV +V | 1,238 (0.4%) | 58 (6.1%) |
| **CCI (original)** |  |  |
| Primary episode |  |  |
| 0 | 189,144 (69%) | 309 (32%) |
| 1 | 59,604 (22%) | 245 (26%) |
| 2 | 17,763 (6.4%) | 133 (14%) |
| 3+ | 9,129 (3.3%) | 267 (28%) |
| 1-year lead-up |  |  |
| 0 | 182,353 (66%) | 285 (30%) |
| 1 | 60,746 (22%) | 239 (25%) |
| 2 | 20,318 (7.4%) | 133 (14%) |
| 3+ | 12,223 (4.4%) | 297 (31%) |
| 2-year lead-up |  |  |
| 0 | 177,293 (64%) | 269 (28%) |
| 1 | 61,142 (22%) | 222 (23%) |
| 2 | 22,404 (8.1%) | 133 (14%) |
| 3+ | 14,801 (5.4%) | 330 (35%) |
| 5-year lead-up |  |  |
| 0 | 168,611 (61%) | 242 (25%) |
| 1 | 61,452 (22%) | 214 (22%) |
| 2 | 25,967 (9.4%) | 142 (15%) |
| 3+ | 19,610 (7.1%) | 356 (37%) |
| All episodes |  |  |
| 0 | 157,602 (57%) | 215 (23%) |
| 1 | 60,880 (22%) | 199 (21%) |
| 2 | 31,222 (11%) | 145 (15%) |
| 3+ | 25,936 (9.4%) | 395 (41%) |
| **CCI (SHMI)** |  |  |
| Primary episode | 0.0 (0.0, 4.0) | 5.0 (0.0, 14.0) |
| 1-year lead-up | 0.0 (0.0, 4.0) | 7.0 (0.0, 14.0) |
| 2-year lead-up | 0.0 (0.0, 4.0) | 8.0 (0.0, 15.0) |
| 5-year lead-up | 0.0 (0.0, 4.0) | 9.0 (3.0, 17.0) |
| All episodes | 0.0 (0.0, 7.0) | 11.0 (3.0, 18.0) |
| **Elixhauser** |  |  |
| Primary episode | 0.0 (0.0, 1.0) | 5.0 (0.0, 11.0) |
| 1-year lead-up | 0.0 (0.0, 3.0) | 5.0 (0.0, 12.0) |
| 2-year lead-up | 0.0 (0.0, 3.0) | 5.0 (0.0, 12.0) |
| 5-year lead-up | 0.0 (0.0, 3.0) | 5.0 (0.0, 13.0) |
| All episodes | 0.0 (0.0, 3.0) | 6.0 (0.0, 14.0) |
| **Frailty** |  |  |
| Primary episode | 0.00 (0.00, 1.30) | 1.50 (0.00, 4.30) |
| 1-year lead-up | 0.00 (0.00, 1.50) | 2.20 (0.00, 6.00) |
| 2-year lead-up | 0.00 (0.00, 1.80) | 2.60 (0.33, 6.70) |
| 5-year lead-up | 0.50 (0.00, 2.30) | 3.10 (0.90, 7.70) |
| All episodes | 1.30 (0.00, 3.20) | 3.90 (1.40, 8.67) |
| ^1^Statistics presented: median (IQR); n (%) | | |
