# Supplementary material for: A comparison of comorbidity measures for predicting mortality after elective hip and knee replacement: A cohort study of data from the National Joint Registry in England and Wales
Source: PLoS One. 2021 Aug 12;16(8):e0255602. doi: 10.1371/journal.pone.0255602 (PMC8360555; doi:10.1371/journal.pone.0255602)
Supplement: S3 Table — (DOCX) [file pone.0255602.s003.docx]

S3 Table: A comparison of the comorbidity scores of people having a primary KR who died within 90-days of their operation and those who were alive at 90-days

| Characteristic | **Alive at 90 days** | **Died by 90 days** |
| --- | --- | --- |
|  | N = 337,417^1^ | N = 870^1^ |
| **ASA Grade** |  |  |
| I | 31,924 (9.5%) | 23 (2.6%) |
| II | 249,274 (74%) | 485 (56%) |
| III | 55,227 (16%) | 335 (39%) |
| IV +V | 992 (0.3%) | 27 (3.1%) |
| **CCI (original)** |  |  |
| Primary episode |  |  |
| 0 | 218,807 (65%) | 349 (40%) |
| 1 | 83,976 (25%) | 215 (25%) |
| 2 | 23,702 (7.0%) | 139 (16%) |
| 3+ | 10,932 (3.2%) | 167 (19%) |
| 1-year lead-up |  |  |
| 0 | 210,398 (62%) | 320 (37%) |
| 1 | 85,551 (25%) | 221 (25%) |
| 2 | 26,971 (8.0%) | 139 (16%) |
| 3+ | 14,497 (4.3%) | 190 (22%) |
| 2-year lead-up |  |  |
| 0 | 203,429 (60%) | 297 (34%) |
| 1 | 86,310 (26%) | 224 (26%) |
| 2 | 29,858 (8.8%) | 139 (16%) |
| 3+ | 17,820 (5.3%) | 210 (24%) |
| 5-year lead-up |  |  |
| 0 | 191,601 (57%) | 272 (31%) |
| 1 | 86,637 (26%) | 209 (24%) |
| 2 | 35,132 (10%) | 137 (16%) |
| 3+ | 24,047 (7.1%) | 252 (29%) |
| All episodes |  |  |
| 0 | 177,940 (53%) | 239 (27%) |
| 1 | 85,902 (25%) | 195 (22%) |
| 2 | 41,414 (12%) | 151 (17%) |
| 3+ | 32,161 (9.5%) | 285 (33%) |
| **CCI (SHMI)** |  |  |
| Primary episode | 0.0 (0.0, 4.0) | 4.0 (0.0, 11.0) |
| 1-year lead-up | 0.0 (0.0, 4.0) | 4.0 (0.0, 13.0) |
| 2-year lead-up | 0.0 (0.0, 4.0) | 4.0 (0.0, 13.0) |
| 5-year lead-up | 0.0 (0.0, 4.0) | 7.0 (0.0, 15.0) |
| All episodes | 0.0 (0.0, 7.0) | 8.0 (0.0, 17.0) |
| **Elixhauser** |  |  |
| Primary episode | 0.0 (0.0, 1.0) | 3.0 (0.0, 7.0) |
| 1-year lead-up | 0.0 (0.0, 2.0) | 3.0 (0.0, 8.0) |
| 2-year lead-up | 0.0 (0.0, 3.0) | 3.0 (0.0, 8.0) |
| 5-year lead-up | 0.0 (0.0, 3.0) | 4.0 (0.0, 9.0) |
| All episodes | 0.0 (0.0, 3.0) | 4.0 (0.0, 10.0) |
| **Frailty** |  |  |
| Primary episode | 0.00 (0.00, 1.10) | 1.40 (0.00, 4.00) |
| 1-year lead-up | 0.00 (0.00, 1.50) | 1.60 (0.00, 4.70) |
| 2-year lead-up | 0.00 (0.00, 1.80) | 2.00 (0.00, 5.40) |
| 5-year lead-up | 0.50 (0.00, 2.30) | 2.80 (0.40, 6.80) |
| All episodes | 1.50 (0.00, 3.40) | 3.70 (1.30, 8.20) |
| ^1^Statistics presented: median (IQR); n (%) | | |
