# Supplementary material for: A comparison of comorbidity measures for predicting mortality after elective hip and knee replacement: A cohort study of data from the National Joint Registry in England and Wales
Source: PLoS One. 2021 Aug 12;16(8):e0255602. doi: 10.1371/journal.pone.0255602 (PMC8360555; doi:10.1371/journal.pone.0255602)
Supplement: S4 Table — (DOCX) [file pone.0255602.s004.docx]

S4 Table: The area under the ROC curve and IPA scores from each of the 5 cross-validation folds for ASA grade and all comorbidity scores for models of 90-day mortality after THR, adjusted for age and gender.

| Characteristic | AUC | | | | | IPA | | | | |
| --- | --- | --- | --- | --- | --- | --- | --- | --- | --- | --- |
|  | Fold 1 | Fold 2 | Fold 3 | Fold 4 | Fold 5 | Fold 1 | Fold 2 | Fold 3 | Fold 4 | Fold 5 |
| **Base** | 0.758 | 0.701 | 0.741 | 0.692 | 0.707 | -0.1100 | 0.0646 | 0.0394 | -0.0713 | 0.0955 |
| **ASA Grade** | 0.811 | 0.750 | 0.793 | 0.740 | 0.785 | -0.1045 | 0.0673 | 0.0440 | -0.0715 | 0.0983 |
| **CCI (original)** |  |  |  |  |  |  |  |  |  |  |
| Primary episode | 0.827 | 0.766 | 0.821 | 0.783 | 0.814 | -0.1018 | 0.0677 | 0.0441 | -0.0667 | 0.1000 |
| 1-year lead-up | 0.824 | 0.774 | 0.816 | 0.777 | 0.818 | -0.1026 | 0.0682 | 0.0440 | -0.0681 | 0.0994 |
| 2-year lead-up | 0.826 | 0.783 | 0.813 | 0.776 | 0.822 | -0.1027 | 0.0678 | 0.0436 | -0.0681 | 0.1002 |
| 5-year lead-up | 0.818 | 0.787 | 0.806 | 0.769 | 0.818 | -0.1043 | 0.0681 | 0.0424 | -0.0682 | 0.0993 |
| All episodes | 0.814 | 0.783 | 0.797 | 0.761 | 0.811 | -0.1048 | 0.0669 | 0.0421 | -0.0682 | 0.0993 |
| **CCI (SHMI)** |  |  |  |  |  |  |  |  |  |  |
| Primary episode | 0.830 | 0.771 | 0.826 | 0.778 | 0.809 | -0.0996 | 0.0632 | 0.0348 | -0.0599 | 0.1054 |
| 1-year lead-up | 0.827 | 0.775 | 0.818 | 0.772 | 0.807 | -0.1045 | 0.0612 | 0.0377 | -0.0642 | 0.0998 |
| 2-year lead-up | 0.830 | 0.778 | 0.812 | 0.767 | 0.809 | -0.1048 | 0.0638 | 0.0319 | -0.0647 | 0.1002 |
| 5-year lead-up | 0.822 | 0.775 | 0.804 | 0.758 | 0.803 | -0.1062 | 0.0648 | 0.0367 | -0.0659 | 0.1000 |
| All episodes | 0.815 | 0.773 | 0.796 | 0.752 | 0.796 | -0.1060 | 0.0649 | 0.0382 | -0.0672 | 0.1003 |
| **Elixhauser** |  |  |  |  |  |  |  |  |  |  |
| Primary episode | 0.834 | 0.767 | 0.822 | 0.796 | 0.822 | -0.0881 | 0.0771 | 0.0541 | -0.0467 | 0.1106 |
| 1-year lead-up | 0.831 | 0.784 | 0.822 | 0.794 | 0.822 | -0.0983 | 0.0705 | 0.0510 | -0.0512 | 0.0986 |
| 2-year lead-up | 0.837 | 0.794 | 0.819 | 0.799 | 0.829 | -0.1071 | 0.0708 | 0.0461 | -0.0554 | 0.0950 |
| 5-year lead-up | 0.834 | 0.805 | 0.818 | 0.793 | 0.824 | -0.1038 | 0.0677 | 0.0446 | -0.0645 | 0.0961 |
| All episodes | 0.832 | 0.802 | 0.813 | 0.787 | 0.820 | -0.1067 | 0.0674 | 0.0459 | -0.0648 | 0.0974 |
| **Frailty** |  |  |  |  |  |  |  |  |  |  |
| Primary episode | 0.815 | 0.726 | 0.781 | 0.738 | 0.764 | -0.0969 | 0.0647 | 0.0366 | -0.0648 | 0.0936 |
| 1-year lead-up | 0.813 | 0.738 | 0.788 | 0.740 | 0.767 | -0.1043 | 0.0678 | 0.0365 | -0.0732 | 0.0903 |
| 2-year lead-up | 0.815 | 0.738 | 0.786 | 0.737 | 0.770 | -0.1055 | 0.0674 | 0.0334 | -0.0715 | 0.0898 |
| 5-year lead-up | 0.813 | 0.736 | 0.782 | 0.730 | 0.772 | -0.1079 | 0.0666 | 0.0348 | -0.0734 | 0.0928 |
| All episodes | 0.809 | 0.731 | 0.781 | 0.729 | 0.770 | -0.1071 | 0.0672 | 0.0365 | -0.0726 | 0.0942 |
