# Supplementary material for: A comparison of comorbidity measures for predicting mortality after elective hip and knee replacement: A cohort study of data from the National Joint Registry in England and Wales
Source: PLoS One. 2021 Aug 12;16(8):e0255602. doi: 10.1371/journal.pone.0255602 (PMC8360555; doi:10.1371/journal.pone.0255602)
Supplement: S5 Table — (DOCX) [file pone.0255602.s005.docx]

S5 Table: The area under the ROC curve and IPA scores from each of the 5 cross-validation folds for ASA grade and all comorbidity scores for models of 90-day mortality after KR, adjusted for age and gender.

| Characteristic | AUC | | | | | IPA | | | | |
| --- | --- | --- | --- | --- | --- | --- | --- | --- | --- | --- |
|  | Fold 1 | Fold 2 | Fold 3 | Fold 4 | Fold 5 | Fold 1 | Fold 2 | Fold 3 | Fold 4 | Fold 5 |
| **Base** | 0.757 | 0.745 | 0.744 | 0.761 | 0.721 | 0.1126 | 0.0210 | -0.0873 | 0.0272 | -0.0545 |
| **ASA Grade** | 0.772 | 0.768 | 0.767 | 0.784 | 0.743 | 0.1159 | 0.0213 | -0.0850 | 0.0284 | -0.0524 |
| **CCI (original)** |  |  |  |  |  |  |  |  |  |  |
| Primary episode | 0.782 | 0.784 | 0.775 | 0.785 | 0.755 | 0.1139 | 0.0254 | -0.0829 | 0.0304 | -0.0523 |
| 1-year lead-up | 0.780 | 0.781 | 0.777 | 0.786 | 0.757 | 0.1137 | 0.0246 | -0.0833 | 0.0295 | -0.0519 |
| 2-year lead-up | 0.778 | 0.780 | 0.780 | 0.784 | 0.760 | 0.1138 | 0.0242 | -0.0839 | 0.0293 | -0.0522 |
| 5-year lead-up | 0.778 | 0.779 | 0.780 | 0.778 | 0.760 | 0.1143 | 0.0240 | -0.0845 | 0.0292 | -0.0519 |
| All episodes | 0.780 | 0.773 | 0.777 | 0.776 | 0.764 | 0.1139 | 0.0230 | -0.0848 | 0.0286 | -0.0522 |
| **CCI (SHMI)** |  |  |  |  |  |  |  |  |  |  |
| Primary episode | 0.785 | 0.784 | 0.783 | 0.794 | 0.765 | 0.1126 | 0.0282 | -0.0785 | 0.0333 | -0.0468 |
| 1-year lead-up | 0.780 | 0.780 | 0.785 | 0.791 | 0.768 | 0.1139 | 0.0271 | -0.0811 | 0.0297 | -0.0458 |
| 2-year lead-up | 0.777 | 0.777 | 0.789 | 0.785 | 0.769 | 0.1145 | 0.0232 | -0.0820 | 0.0274 | -0.0481 |
| 5-year lead-up | 0.782 | 0.778 | 0.790 | 0.779 | 0.772 | 0.1153 | 0.0231 | -0.0802 | 0.0272 | -0.0485 |
| All episodes | 0.784 | 0.771 | 0.784 | 0.778 | 0.777 | 0.1148 | 0.0225 | -0.0807 | 0.0288 | -0.0501 |
| **Elixhauser** |  |  |  |  |  |  |  |  |  |  |
| Primary episode | 0.790 | 0.781 | 0.775 | 0.787 | 0.767 | 0.1143 | 0.0341 | -0.0783 | 0.0316 | -0.0503 |
| 1-year lead-up | 0.787 | 0.780 | 0.776 | 0.783 | 0.770 | 0.1131 | 0.0301 | -0.0807 | 0.0308 | -0.0509 |
| 2-year lead-up | 0.786 | 0.779 | 0.784 | 0.782 | 0.772 | 0.1120 | 0.0294 | -0.0819 | 0.0294 | -0.0519 |
| 5-year lead-up | 0.787 | 0.778 | 0.779 | 0.777 | 0.769 | 0.1119 | 0.0276 | -0.0830 | 0.0298 | -0.0526 |
| All episodes | 0.785 | 0.774 | 0.773 | 0.774 | 0.769 | 0.1116 | 0.0255 | -0.0840 | 0.0294 | -0.0516 |
| **Frailty** |  |  |  |  |  |  |  |  |  |  |
| Primary episode | 0.782 | 0.782 | 0.772 | 0.792 | 0.759 | 0.1073 | 0.0307 | -0.0805 | 0.0201 | -0.0519 |
| 1-year lead-up | 0.779 | 0.781 | 0.773 | 0.791 | 0.757 | 0.1066 | 0.0239 | -0.0830 | 0.0162 | -0.0517 |
| 2-year lead-up | 0.777 | 0.778 | 0.775 | 0.790 | 0.757 | 0.1087 | 0.0187 | -0.0842 | 0.0186 | -0.0529 |
| 5-year lead-up | 0.775 | 0.776 | 0.772 | 0.789 | 0.756 | 0.1049 | 0.0217 | -0.0842 | 0.0233 | -0.0518 |
| All episodes | 0.774 | 0.775 | 0.767 | 0.788 | 0.756 | 0.1060 | 0.0234 | -0.0842 | 0.0233 | -0.0522 |
