# Supplementary material for: A comparison of comorbidity measures for predicting mortality after elective hip and knee replacement: A cohort study of data from the National Joint Registry in England and Wales
Source: PLoS One. 2021 Aug 12;16(8):e0255602. doi: 10.1371/journal.pone.0255602 (PMC8360555; doi:10.1371/journal.pone.0255602)

S3 Fig: Histograms comparing the distribution of Elixhauser comorbidity scores (blue) calculated over different lead-up times, compared with using all episodes (grey)


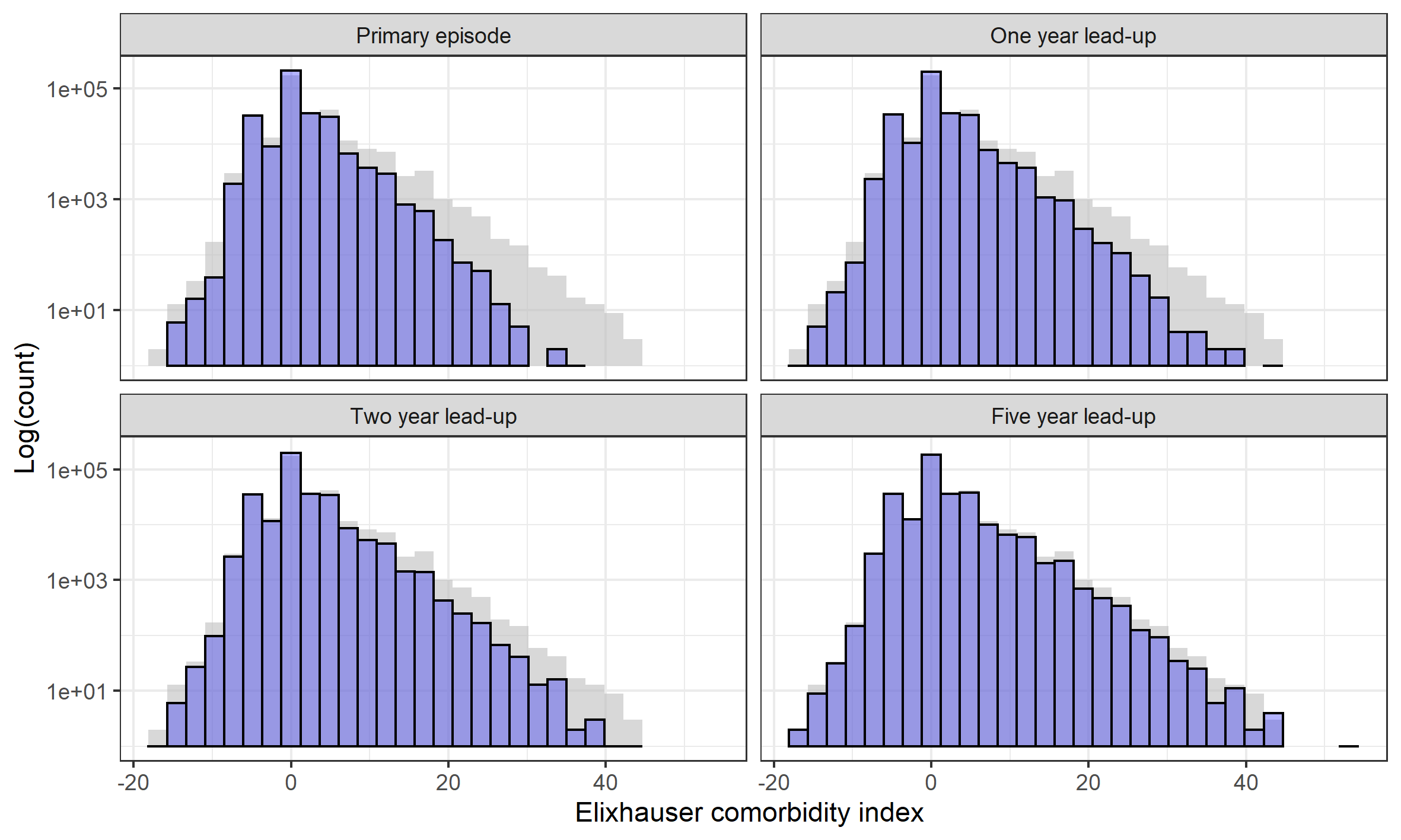

Supplement: S3 Fig — Histograms comparing the distribution of Elixhauser comorbidity scores (blue) calculated over different lead-up times, compared with using all episodes (grey). (DOCX) [file pone.0255602.s008.docx]
