# Supplementary material for: A comparison of comorbidity measures for predicting mortality after elective hip and knee replacement: A cohort study of data from the National Joint Registry in England and Wales
Source: PLoS One. 2021 Aug 12;16(8):e0255602. doi: 10.1371/journal.pone.0255602 (PMC8360555; doi:10.1371/journal.pone.0255602)

S5 Fig: A comparison of ROC curves from logit models of 90-day mortality after primary THR and ASA + Charlson Comorbidity scores derived using different lead-up periods


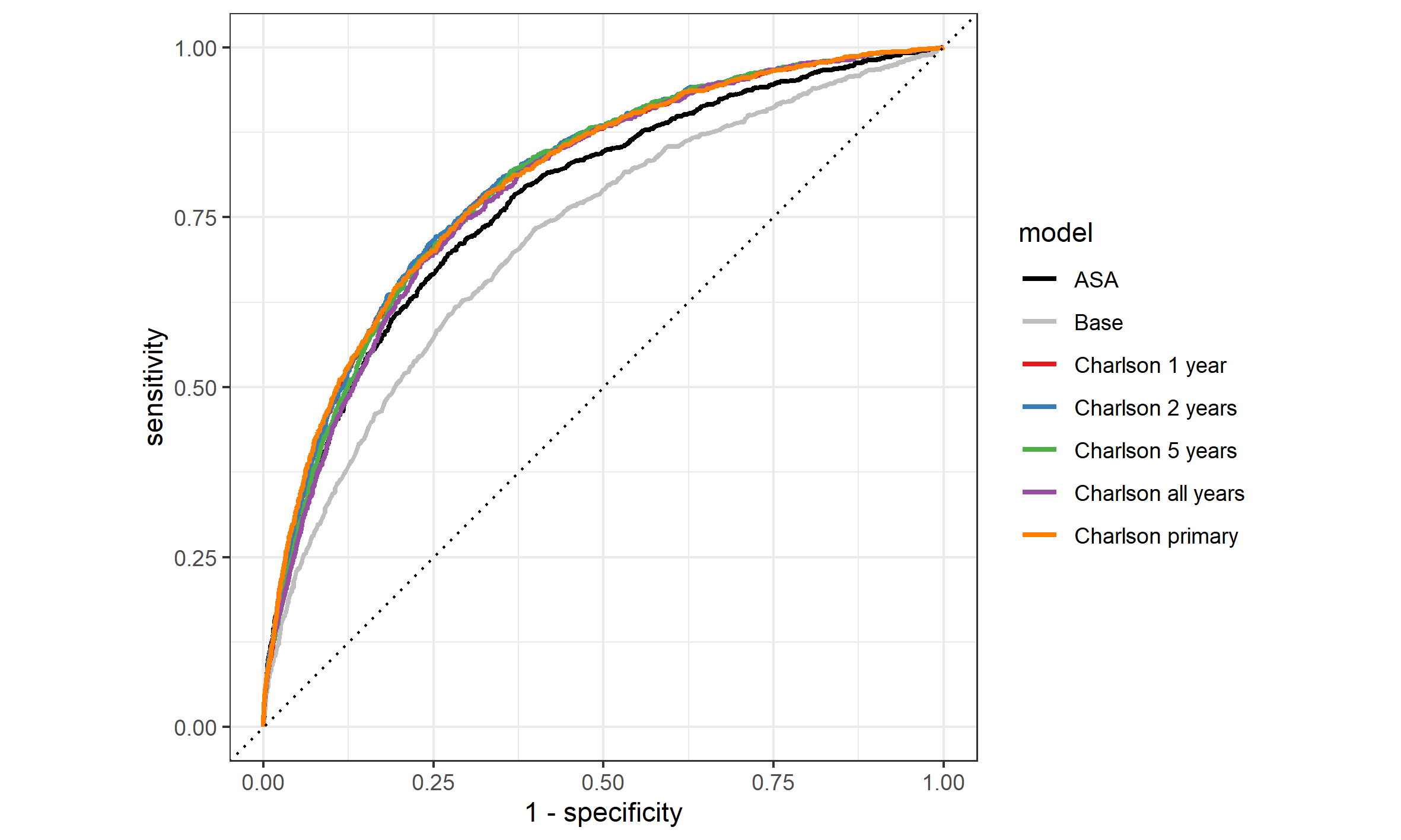

Supplement: S5 Fig — (DOCX) [file pone.0255602.s010.docx]
