# Supplementary material for: A comparison of comorbidity measures for predicting mortality after elective hip and knee replacement: A cohort study of data from the National Joint Registry in England and Wales
Source: PLoS One. 2021 Aug 12;16(8):e0255602. doi: 10.1371/journal.pone.0255602 (PMC8360555; doi:10.1371/journal.pone.0255602)
Supplement: Attachment — Submitted filename: response to reviewers.docx [file pone.0255602.s020.docx]

**Response to reviewers’ comments on manuscript # PONE-D-21-03064**

18^th^ June 2021

Dear Chief Editor,

Please find enclosed our revised manuscript, entitled **A comparison of comorbidity measures for predicting mortality after elective hip and knee replacement: A cohort study of data from the National Joint Registry in England and Wales**.

We thank the reviewers for their careful review of our article. They have highlighted important areas in which our article can be improved. We have responded in red to each comment below.

We look forward to hearing from you at your earliest convenience,

Yours sincerely,

Dr Chris Penfold

MSci, PhD

**Reviewer #1:**

1. It might be interesting to compare the models with a base model only. Hence, a model with age and sex only. I suspect that such model would not perform much worse than model 1?

We have updated our analyses to include the base model (page 5, line 145), adjusted for age and gender only. We have also included the base model in our comparisons of model performance (page 7, lines 199-200).

2. I am a little skeptical to the use of the Brier score. It is a common measure of discriminatory ability, but it is actually quite insensitive for rare events data, as seen from the tables where this metric is almost constant. I would consider omitting this metric. A near-by-alternative might be the Index of Prediction Accuracy (IPA) instead. It is still based on the Brier score but with a better scale.

Thank you for this suggestion. We were not familiar with the Index of Prediction Accuracy. We have replaced the Brier score with the IPA and amended the relevant text in the Methods (page 6, lines 153-156) and Results (page 7, lines 200-204 and 209-214).

3. I agree that external validation seems to be outside the scope of the article. The data set is quite big, however, so I am just wondering whether it wouldn’t be possible to save a small portion from the original data for later assessment?

We have opted to use 5-fold cross-validation and have updated our Methods (page 5, lines 156-157) and Results to reflect this change. In the Results section we have included details of the change in AUC and IPA estimates within each of the 5 folds (page 8, lines 225-229 and Tables S4 and S5).

4. You should probably discuss if there was any (and if so, how much) censoring, especially for the one-year time frame. If relevant, you might discuss why you did not consider a time-dependent ROC-curve.

We assume the reviewer is specifically referring to censoring due to missing dates of deaths. Since our mortality outcome is recorded through centrally collected civil registration records, missing dates of deaths are very unlikely to occur for people still resident in the UK when they died. However, it is plausible that the recording of dates of deaths may be delayed or missed entirely for people who died after emigrating from the UK. Censoring due to loss-to-follow-up of this type has not been quantified for the NJR, but we anticipate it affecting only a small number of people and consequently having only a negligible effect on our study. We have acknowledged this as a weakness of our study (page 9, lines 272-276).

5. I would suggest a sensitivity analysis without stratification for the bootstrap replicates used for the confidence intervals. I think you should have a reasonably good chance to succeed without it (which would be a strength).

We thank the reviewer for this suggestion. Rather than include this as a sensitivity analysis we have changed our analysis approach and updated our Methods to specify that confidence intervals be calculated using the exact method to evaluate the uncertainty of AUC developed by DeLong using the algorithm proposed by Sun and Xu in the ‘pROC’ package (page 6, lines 163-165).

Smaller issues

----------------

1. The use of logistic regression might be a little problematic. Nevertheless, I would use it myself (I am not saying I know a better method!), but it might be discussed as a limitation that this method is actually not recommended for very rare outcomes (which is what we have here).

We have erred on the side of ‘tried and tested’ in our use of logistic regression, but we will keep abreast of developments in methods for very rare outcomes for future work.

We have amended the Discussion as follows (page 10, line 298):

“Future research may explore whether these models can be improved by using other algorithms in addition to logit models, particularly for very rare outcomes such as mortality after elective arthroplasty.”

2. I am lacking some details of how you treated patients with surgeries performed on multiple joints. They can only die once I think.

A small number of patients had a second primary operation (contralateral primary) in the follow-up timeframe. We have included a sensitivity analysis in which the first primary operation for patients who had contralateral primaries was censored at the time of the 2^nd^ primary. We have updated the Methods (page 5, lines 159-161) and Results (page 8, lines 230-232) with the relevant details. Outcomes were unaffected by this sensitivity analysis.

3. Is it reasonable that patients with ASA class V get elective surgery?

We agree that it is extremely unlikely that a patient with ASA class V would have elective surgery. A potential explanation is a simple data entry error (ASA class IV was intended). For this reason, and due to the extremely low number of people classed as ASA V, we combined ASA classes IV and V in our analyses. We have clarified this in our Methods (page 4, line 116).

4. You refer to the comorbidity indices as being “continuous”. I guess this might be a quite reasonable approximation for the Elixhauser score (which is on a discrete scale from 0 to 30). I am less convinced for CCI, however, since it is a weighted sum with only a limited set of possible values.

We have now included CCI as a categorical variable in our models and updated the Methods (page 4, lines 121) and Results to reflect this.

5. There has been some (relatively recent and perhaps valid) criticism concerning the weights used in CCI. Might be worth considering? See <https://www.jclinepi.com/article/S0895-4356(16)30676-X/pdf>

We have included this as a discussion point. Given that the focus of our study is on whether comorbidity scores as they are currently used offer any advantage over ASA grade we feel it is beyond the scope of this article to focus on the correct mathematical handling of the weights used in CCI. However, we anticipate this will be an important area of work in the future. We have amended our Discussion to include the following (page 10, lines 299-304):

“Although we have used the comorbidity indices as they have been used in many other studies, the additive approach used to combine conditions in the CCI is algebraically incorrect (25) and Elixhauser et al. intended the comorbidities to be retained as independent measures rather than used to derive a summary Elixhauser index (8). It may therefore be valuable to explore the impact of these accepted but incorrect approaches may have on mortality prediction.”

6. It might be noted that Elixhauser herself did not recommend using her tool as a combined measure. Therefore, it might be relevant to point out that what is not working, is to use her tool as is commonly done in practice, although usage according to her original suggestion might still work (which is usually not possible, however, due to limited data and the need of too many variables).

Please see the amendment to our Discussion summarised above (point 5).

7. I would prefer to have some data on ASA class (and perhaps diagnosis) already in table 1 and 2 (it is now in the supplement).

We have included ASA grade in Tables 1 and 2.

8. It might be beneficial to instead include some additional data concerning individual comorbidities (according to CCI/Elixhauser) in the supplement. It might be slightly outside the scope of the article, but I do think it would be interesting to be able to compare conditions from the UK to similar data from other countries (as from other papers).

In Tables 1 and 2 we have included the prevalence of all the conditions which are used in the CCI and Elixhauser Index. We have described these in our Results (pages 6 & 7, lines 174-185), including highlighting similarities and differences in patients who die within 90 days of THA or KA.

Tiny issues

----------

9. Abstract: I propose to describe the data as “linked” rather than “combined”.

We have replaced ‘combined’ with ‘linked’ in the Abstract.

10. Abstract: “Mortality _after_ 90 days”. Shouldn’t this be “Mortality _within_ 90 days”?

Thanks! We have corrected this.

11. I am not a native English speaker (and I know you are) but for me “sex” would sound more accurate than “gender”, since you use administrative register data. Or were the patients surveyed for gender identity? (You do actually have “sex” in fig 5.)

Thank you for identifying this inconsistency. The NJR data collection form refers to gender, we have therefore used this in our article. We have checked and corrected the text, plots and tables where necessary.

12. I thought ASA was a “class” (not a “grade”)? (I am not sure of the difference myself but this is what I have been told).

In all honesty we aren’t sure whether ASA should be referred to as a grade or class. The unabbreviated form of ASA grade/class is “ASA physical status classification system” which suggests it should be ASA class, but the National Institute for Health and Clinical Excellence (NICE), and many other professional bodies including the NJR, refers to ‘ASA grade’. Given the lack of consensus on this, we hope the reviewer won’t mind us keeping this as ‘ASA grade’ since this is in agreement with the NJR data collection form. We are happy to include this change if necessary.

13. Background: Isn’t Guernsey part of the register (NJR) name (it is mentioned later).

We have updated the Background to include the States of Guernsey.

14. Background: “the main focus within joint replacement surgery has been on the Charlson Comorbidity (CCI) and Elixhauser indices”. I think the RxRisk score has been used quite a bit as well (at least in Australia). Could perhaps be mentioned?

Thanks for highlighting this. We agree that RxRisk score has been used in joint replacement surgery, but it is a prescription rather than diagnosis-based score. We have revised the wording in our Background to specify that the CCI and Elixhauser index are widely used diagnosis-based summary scores.

15. You state that the “original” Elixhauser includes 30 conditions. This is true but since it originally included 31, it might be a little confusing what is actually meant by “original” (one condition was dropped in an update made in 2004).

We thank the reviewer for this comment. We have checked the 1998 paper by Elixhauser et al (cited in our article), which to our knowledge is the original source for the Elixhauser index, and it refers to 30 conditions. We may have overlooked an earlier article and we would be very happy to update this description if the reviewer can direct us to the relevant literature.

16. Methods: Was “HES” used for Wales as well?

HES data are only available for England. We have clarified this within our definition of the study sample (page 4, lines 107-108).

17. Methods: I think that what you refer to as “predictors” might be considered “potential predictors” or similar?

We have replaced “predictors” with “potential predictors”.

18. You might spell out the meaning of “ICD-10” the first time you use this acronym.

We have included ICD-10 in our list of abbreviations. Sorry for this oversight.

19. Predictors section: What is meant by “All available episodes up to the primary”. I suppose that the start of HES indicates a lower limit. When was that?

We have updated our Methods to clarify that HES records are available from 1989 onwards (page 4, line 98).

20. There is a mention of a Brier score of “0.0.0034” (two decimal marks).

We have replaced the Brier score with the IPA.

21. You mention as a strength that you do not need to rely on data that is older than 10 years (and I agree!). But you do in fact include data from early 2011 (which strictly speaking was more than ten years ago).

We have amended the wording as follows (page 8, line 246):

“The size of the NJR meant that we were able to use a more recent dataset and not rely on the outcomes of operations performed **early in the NJR** which may not reflect the current postoperative mortality trends, while still having sufficient events to be confident in our findings.”

22. You mention that HES data from private hospitals were not included and that those patients might be healthier than other patients. I agree, but I also suppose that in some other countries, private hospitals might also have other incitements to report additional comorbidities due to reimbursement? But I suppose you do not have that issue in the UK?

We are not aware of evidence to support incitement to report additional comorbidities in the UK and have been looking into this in related work. We have amended our discussion of weaknesses to include this possibility alongside other discussions around the recording of comorbidities in HES (page 9, line 260).

23. Figure 1 and 2: I would prefer dates as “2011-01-01” (month before day since I think this is more common in other countries.

We have amended the date format in Figures 1 and 2

24. Table 1 and 2 have some superscripts which are not explained.

Thank you and sorry for this oversight. We have included footnotes to explain the superscripts in Tables 1 and 2.

25. For figures S5 etc I think you should clarify that age and sex were included in the models (otherwise the ROC curves should be less smooth).

We have amended the figure titles to include reference to age and gender being in the models.

26. I think you have too many digits in your confidence intervals for AUC. If you have “only” 2000 bootstrap replicates you should only include one significant digit. If you need two digits, you are recommended to have 10000 replicates (according to the documentation for the pROC R package).

Given the revised calculation of the confidence intervals in response to point 5 in the ‘Main issues’ we have retained the current number of significant figures for the confidence intervals.

27. I would recommend including pagination and line numbers for the sake of the reviewers :-)

Sorry for omitting these in the original submission. We’ve added them in to the revised version, which will hopefully make life a bit easier!

**Reviewer #2:**

1. I think it would be better and more consistent to use arthroplasty instead of replacement consistently throughout the whole article and consequently using the abbreviations THA and TKA rather than using both terms at different sections of the article.

Thank you for highlighting this inconsistency. The NJR uses replacement rather than arthroplasty and since we are specifically referring to joint replacements rather than other procedures captured by arthroplasty we have updated our article to use replacement throughout.

2. I would suggest pointing out the most commonly encountered specific comorbidities which might have been correlating / predicting mortality after THA or TKA ...It would have ideal to include them in the analysis in addition to the use of the whole scores parameters.

We have included the prevalence of the underlying conditions used to calculate the Charlson and Elixhauser scores in Tables 1 and 2. We have also described the most prevalent comorbidities in patients who died within 90 days of their primary THR or KR (pages 6 & 7, lines 174-185). We agree that it may be interesting to include the individual conditions in our analyses, particularly to explore their validity in predicting mortality risk for arthroplasty patients. However, the focus of this article is on the use of existing comorbidity scores compared with ASA grade. Reviewer 1 raised the point that Elixhauser did not intend for individual comorbidities to be combined into a summary score. We have included exploring the impact of retaining individual comorbidities for predicting mortality risk as an area for future research (page 10, lines 299-304).
